# Managing Spontaneous Abortion Hemorrhage: An Adult Simulation Case for Emergency Medicine Residents

**DOI:** 10.5070/M5.52263

**Published:** 2026-07-31

**Authors:** Brendan Freeman, Ritika Gudhe, Rodrigo Kong, Eric Levy, Anand Swaminathan

**Affiliations:** * University of Massachusetts Chan Medical School, Department of Emergency Medicine, Worcester, MA; ^ Staten Island University Hospital/Northwell Health, Department of Emergency Medicine, Staten Island, NY

## Abstract

**Audience:**

This simulation is intended for emergency medicine residents, PGY 1–4.

**Introduction:**

Postpartum hemorrhage (PPH) is defined as cumulative blood loss ≥1000 mL or any postpartum bleeding with signs of hypovolemia within 24 hours of delivery. Post-abortion hemorrhage (PAH) has no single universally adopted numeric definition but clinically is defined as excessive bleeding following spontaneous or induced abortion that results in hemodynamic instability, need for transfusion, hospital admission, or procedural intervention. Due to its rarity, PAH may be underrecognized and thus is the target of this simulation. The emergency department is the country's reproductive health safety net. With the decline of labor and delivery units and on-call, in-house OB/GYN consultants, emergency clinicians must be equipped to recognize and manage these cases.^1^ Effective management of these cases includes uterotonic medications and uterine tamponade devices like the Bakri balloon.^2^ There is mixed evidence on the use of Bakri balloons for post-abortion hemorrhage, with some hospitals moving towards vacuum-assisted devices (e.g. JADA).^3^ However, they have remained relevant in non-OB settings and institutions with limited obstetrical support.^4^–^6^ This simulation aims to address a gap in training for PAH for emergency medicine residents, focusing on the placement of the Bakri balloon.

**Educational Objectives:**

By the end of this simulation, learners should be able to: 1) recognize signs of post-abortion hemorrhage, 2) manage hemorrhage using appropriate physical maneuvers, uterotonic medications, and specialist consultation, 3) demonstrate proper placement of a Bakri balloon.

**Educational Methods:**

This single-center, high-fidelity simulation was conducted at a tertiary care academic center. Simulation was chosen as the best way to teach this topic because it allows a psychologically safe environment to practice a scenario which involves high-level critical thinking and fine-motor skills that would otherwise degrade under stress during a real patient encounter.

**Research Methods:**

Pre- and post-simulation surveys were administered, including Likert-scale questions on comfort with the management and procedures included, and a multiple-choice knowledge assessment. Twenty-eight individuals completed the pre-simulation learner evaluation, and 31 completed the post-simulation learner evaluation. Likert-scale data was treated as ordinal (1 = strongly disagree, 5 = strongly agree) and analyzed using the Mann-Whitney U Test. The multiple-choice knowledge assessment was analyzed using a paired T-test.

**Results:**

The simulation included 29 emergency medicine residents, four MS3 students, four MS4 students, and three junior EM PA fellows. This case was used eight times during the session. The simulation lasts approximately 20 minutes. There were statistically significant improvements across all measures. On a 5-point Likert scale, comfort in placing a Bakri balloon increased from 2.35 to 3.97 (U = 82, z = 5.34, *P* < 0.01); comfort in managing postpartum/abortion hemorrhage rose from 3.11 to 3.90 (U = 197, z = 3.59, *P* < 0.01), and recognition of hemorrhage signs increased from 3.96 to 4.39 (U = 278, z = 2.36, *P* = 0.02). Mean scores on the multiple-choice knowledge assessment improved from 55.79% to 90.52% (t(18) = −5.23, *P* < 0.001) pre- and post-intervention.

**Discussion:**

This simulation successfully addressed the gap in the educational materials available to address the learning of the recognition and management of PPH/PAH, including the placement of intrauterine balloon devices. The simulation increased learners’ comfort in managing PAH and placing Bakri balloons. While effective for immediate skills/knowledge acquisition, this is a high-acuity, low-occurrence procedure that requires repetition and deliberate practice for more durable learning.

**Topics:**

Postpartum hemorrhage, post-abortion hemorrhage, Bakri balloon, uterine tamponade, simulation training.

## USER GUIDE

**Table:** 

List of Resources:	
Abstract	1
User Guide	3
Instructor Materials	68
Operator Materials	77
Debriefing and Evaluation Pearls	79
Simulation Assessment	83
Appendix A: Pre- and Post-Intervention Learner Evaluation	88

**Learner Audience:**

Medical Students

**Time Required for Implementation:**

**Preparation:** 30 minutes for setup and 30 minutes for instructor pre-briefing. Strongly advise a 30-minute preparatory session 1 month prior.

**Time for case:** 15–20 minutes.

**Time for debriefing:** 20–30 minutes.

**Recommended Number of Learners per Instructor:**

The authors recommend two facilitators and 2–6 learners per group.

**Topics:**

Postpartum hemorrhage, post-abortion hemorrhage, Bakri balloon, uterine tamponade, simulation training.

**Objectives:**

By the end of this case, learners should be able to:

1. Recognize signs and symptoms of post-abortion hemorrhage and understand its most common causes.
2. Manage post-abortion hemorrhage by administering appropriate uterotonic medications and performing necessary physical maneuvers.
3. Demonstrate the correct placement of a Bakri balloon, including identifying major contraindications for its use.

### Linked objectives and methods

#### Objective 1 – Identify signs and symptoms of post-abortion hemorrhage and understand its most common causes

In this simulation, learners are presented with a patient exhibiting signs and symptoms of post-abortion hemorrhage (PAH). They assess the patient’s clinical presentation, guided by realistic vitals and simulated bleeding moulage. This scenario helps learners recognize key indicators of PAH, such as tachycardia, hypotension, and excessive bleeding. Additionally, the multiple-choice knowledge assessment (Appendix A) explicitly emphasizes PAH's signs and symptoms and its most common cause. This is further reinforced during the debriefing, reinforcing learner understanding of the condition.

#### Objective 2 – Manage post-abortion hemorrhage by administering appropriate uterotonic medications and performing necessary physical maneuvers

Learners respond to the simulated hemorrhage by selecting and administering appropriate uterotonic medications and performing hands-on physical maneuvers to control bleeding. This is assessed by critical action #3: administer at least one uterotonic medication. Medication indications and contraindications are emphasized in the debrief and the multiple-choice knowledge assessment (appendix A).

#### Objective 3 – Demonstrate the correct placement of a Bakri balloon, including identifying major contraindications for its use

Critical action #5 aligns with this objective. To lessen cognitive load, a preparatory session demonstrating how to use the Bakri balloon was administered one month prior to the simulation. This included watching the following video: https://www.youtube.com/watch?v=EXUjDyHjb10, and a live demonstration by the instructor on a mannequin. Additionally, learners are guided through each step, if needed, during the simulation. During the debriefing, learners review major contraindications, indications, and placement of the Bakri balloon.

### Recommended pre-reading for instructor

Swaminathan A. Post-partum hemorrhage. REBEL EM. January 15, 2018. Accessed February 27, 2026. https://rebelem.com/post-partum-hemorrhage/

Cook Medical. Bakri balloon for postpartum hemorrhage. YouTube. Published March 27, 2012. Accessed February 27, 2026. https://www.youtube.com/watch?v=EXUjDyHjb10

### Learner responsible content

Must attend preparatory session 1 month in advance, which involves watching this video, https://www.youtube.com/watch?v=EXUjDyHjb10, a demonstration by the instructor on a mannequin, and small-group sessions to practice the skill.

### Results and tips for successful implementation

#### Results

**Figure 1:**
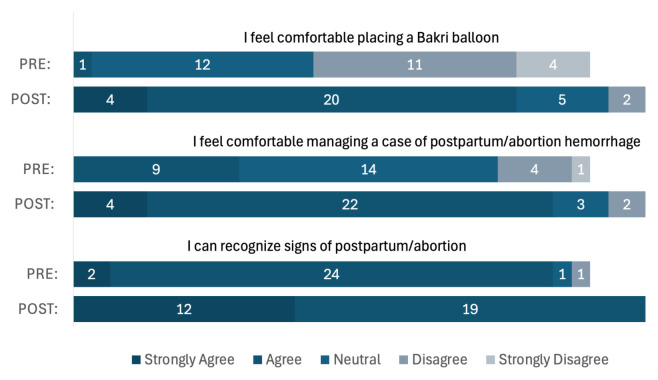
Pre-simulation and post-simulation evaluation survey results of Likert Scale questions which asked learners: “For the following statements, please indicate whether you strongly disagree, disagree, are neutral, agree, or strongly agree. Answer truthfully based on how you really feel at the time of taking this survey.“

We conducted a single-center prospective pre- and post-interventional study evaluating the impact of an educational intervention on EM resident management of a simulated case of PAH. Analysis was conducted by study authors using Microsoft Excel. A *P* value of < 0.05 was considered significant. Kirkpatrick’s levels 1 and 2 were evaluated. Twenty-eight individuals completed the pre-simulation learner evaluation, and 31 completed the post-simulation learner evaluation. Likert-scale data was treated as ordinal (1 = strongly disagree, 5 = strongly agree) and analyzed using the Mann-Whitney U Test. Regarding comfort in placing a Bakri balloon, the mean score increased from 2.35 to 3.97 (*U =* 82, *z =* 5.34, *P* <0.01). Comfort in managing a case of postpartum/abortion hemorrhage had a mean increase from 3.11 to 3.90 (*U* = 197, *z* = 3.59, *P* <0.01), and recognition of signs of postpartum/abortion hemorrhage had a mean increase from 3.96 to 4.39 from pre-simulation to post-simulation (*U* = 278, *z* = 2.36, *P* = 0.02). All increases were statistically significant (Figure 1).

**Figure 2:**
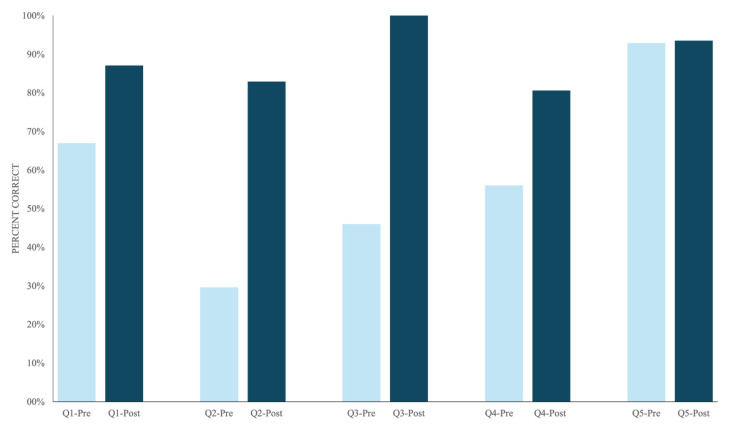
Pre- and post-simulation learner evaluation results of knowledge assessment, which consisted of five multiple choice questions testing knowledge of uterotonic contraindications, maximum volume held by Bakri balloon, the most common cause of postpartum hemorrhage, and contraindications to Bakri balloon placement

Regarding the knowledge assessment portion of the evaluation, of those who completed the assessments, 19 were analyzed using a paired T-test. These were the only individuals who overlapped between the pre-simulation and post-simulation learner evaluations due to clinical scheduling. The scores from these individuals were paired against themselves by matching their user-generated unique anonymous identifiers between pre-simulation and post-simulation responses. There was a statistically significant increase in mean scores of this cohort from pre-simulation to post-simulation of 55.79% to 90.52% (*t*(18) = −5.23, *P* < 0.001). Mean percentage scores were also calculated for all the pre- and post-simulation learner evaluation participants, which are reported in Figure 2.

#### Implementation

Approximately one month prior to the scheduled simulation day, instructors should begin with the provision of a brief 30-minute session on Bakri balloon placement and administer the pre-intervention learner evaluation (appendix A). The preparatory session involves a group viewing of the following video: https://www.youtube.com/watch?v=EXUjDyHjb10, followed by a live demonstration by the instructor on a mannequin. The session is then broken up into small groups where learners can practice the kinesthetic skills of setting up and placing the Bakri balloon. If your facilitator team for the simulation is unfamiliar with this procedure, we strongly advise having them attend this session or hold a dedicated session as well. We recommend keeping this session separate from the simulation day to avoid the priming of learners. Learners should remain blind to the purpose of the session and the pre-intervention evaluation for similar reasons.

One week before the simulation, copies of the case, media, debrief, and Bakri balloon session for review should be emailed to the faculty facilitators. The day prior, the simulation team should prepare the room with all the equipment, case scenarios, and checklists as outlined in this manuscript. We used a Noelle and Victoria Mannequin from Gaumard; however, any mannequin or task-trainer with pelvic access will suffice for this simulation. These mannequins were prepared for the session by placing red-gelatin-soaked Chux under a hospital sheet near the pelvic region. Load stimuli onto your system computer for display when the learner requests it. A QR code to the post-simulation learner evaluations should be printed out for each facilitator group following the debrief. For the authors’ scenario, this preparation took approximately 30 minutes.

A pre-brief should be held prior to the simulation. The author’s team had one 30 minutes prior to the simulation, which involved our review of the case, materials, labs/media, and debrief materials. Two facilitators in our cohort had expertise in Bakri balloon placement. One facilitator was present in each scenario to review this procedure during the debrief for the learners; however, if your team is unfamiliar with this procedure, this should also be reviewed again in the pre-brief. Facilitators should consider assessing learners using the provided critical actions checklist during the simulation below; however, our session did not formally track these for learners.

This simulation occurred in our simulation center and was one case out of a multi-station simulation day that occurs every other month as part of emergency medicine resident education. Residents were allotted approximately 15–20 minutes to complete the case. A debriefing session followed each case and lasted approximately 20–30 minutes. This generally left about 10 minutes between groups to turn over the room. A total of 8 groups participated in this simulation over the course of the day, each containing 5 group members. Groups were assigned the week prior and included one or more residents from PGY 1–3, an emergency medicine physician assistant fellow, and a third-year medical student (MS3) or fourth-year medical student (MS4).

## Figures and Tables

**Table:** 

**#1**	Venous blood gas (VBG)
**#2**	Complete blood count (CBC)
**#3**	Basic metabolic panel (BMP)
**#4**	Liver function tests (LFT)
**#5**	Urinalysis (UA)
**#6**	Coagulation Panel
**#7**	Troponin T
**#8**	Serum Pro BNP
**#9**	Transvaginal ultrasound
**#10**	Electrocardiogram (ECG)

**Table:** 

**Vitals:**	Heart Rate (HR) 123	Blood Pressure (BP) 106/96
	Respiratory Rate (RR) 18	Temperature (T) 98.4°F
	Oxygen Saturation (O_2_Sat) 98%	

**Table:** 

pH	7.4
pCO2 43 mmHg	
pO2	40 mmHg
HCO3	24 mmol/L
SaO2	96%
Lactate	3.5

**Table:** 

White blood count (WBC)	9.0 x 1000/mm^3^
Hemoglobin (Hgb)	5.3 g/dL
Hematocrit (HCT)	20%
Platelet (Plt)	201 x 1000/mm^3^

**Table:** 

Sodium	132 mEq/L
Potassium	4.0 mEq/L
Chloride	100 mEq/L
Bicarbonate (HCO3)	24 mEq/L
Blood Urea Nitrogen (BUN)	14 mg/dL
Creatinine (Cr)	0.9 mg/dL
Glucose	89 mg/dL

**Table:** 

Albumin	4 g/dL
Total Protein	9.8 g/dL
Total bilirubin	1.1 mg/dL
Direct bilirubin	0.2 mg/dL
Alkaline phosphatase	60 units/L
Aspartate aminotransferase (AST)	31 units/L
Alanine aminotransferase (ALT)	29 units/L

**Table:** 

Color	yellow
pH	7
Specific Gravity	1.025
Glucose	negative
Protein	negative
Ketones	negative
Bilirubin	negative
Leukocyte esterase	negative
Nitrites	negative
Blood	0

**Table:** 

Prothrombin Time (PT)	12.0 seconds
Partial Thromboplastin Time (PTT)	31.0 seconds
International Normalized Ratio (INR)	1.0

**Table:** 

Troponin T	<6 ng/mL
Serum Pro BNP	100 pg/mL

**Figure:**
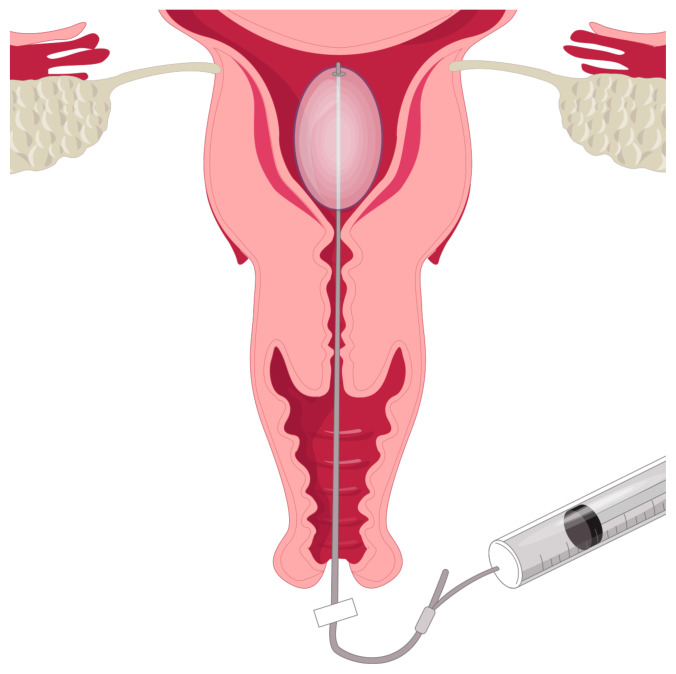


**Table:** SIMULATION EVENTS TABLE:

Minute (state)	Participant action/ trigger	Patient status (simulator response) & operator prompts	Monitor display (vital signs)
Event 1 – Initial Presentation (0:00)	Perform primary survey Establish IV access and place on monitor Obtain focused pregnancy history Perform GU exam Order ultrasound	If pregnancy and hemorrhage are recognized: Ongoing heavy vaginal bleeding Tachycardia persists but patient remains perfusing If pregnancy/hemorrhage not recognized (no GU exam or ultrasound within several minutes): Continued bleeding Progressive tachycardia and hypotension Patient appears increasingly pale and altered	Initial: HR: 123 BP: 106/96 RR: 18 O2: 98% RA If delayed recognition: HR: 160 BP: 82/46
Event 2 – Hemorrhagic Decompensation (5:00)	Administer uterotonics Activate massive transfusion protocol Consult OBGYN	If uterotonics administered: Partial reduction in bleeding Hemodynamics stabilize but remain abnormal If blood products given without uterotonics: Ongoing hemorrhage No meaningful hemodynamic improvement If no hemorrhage control initiated: Rapid deterioration to PEA arrest	Decompensation: HR: 165 BP: 76/42 If uterotonics given: HR: 120 BP: 90/60 If untreated: pulseless electrical activity
Event 3 – Escalation / Mechanical Intervention (10:00)	Placement of Bakri balloon Continued transfusion support	If Bakri balloon placed correctly: Significant reduction in bleeding Hemodynamic improvement If Bakri balloon not placed: Continued instability Recurrent hypotension despite transfusion	After successful placement: HR: 115 BP: 98/52 If not placed: persistent hypotension
Event 3 – Case Completion (15:00)	Admit to OBGYN Prepare for operative management	Hemorrhage controlled Hemodynamically stabilized	HR: 110 BP: 100/70 RR: 18 O2: 98% RA

**Image 1 f3-jetem-11-3-s63:**
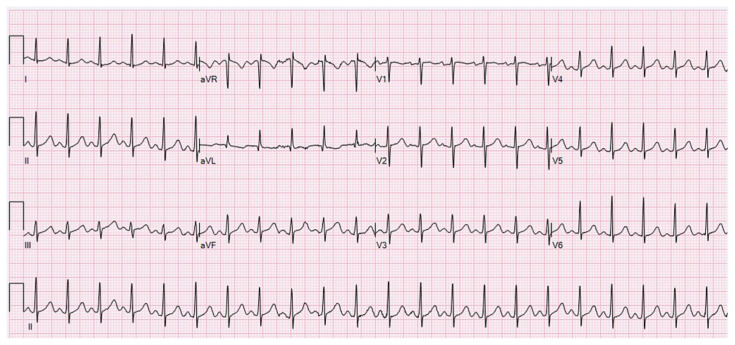
Hariadhi. Bakri Balloon. In: Wikimedia Common. https://commons.wikimedia.org/wiki/File:Bakri_Balloon_Hariadhi.svg Published May 19, 2025. Accessed May 30, 2026. License CC BY-SA 4.0.

**Table:** 

**Critical Actions:** Place two large-bore IVs. Perform a GU exam and obtain a transvaginal ultrasound. Administer at least one uterotonic medication. Consult OBGYN for guidance on further management.	0:00

**Table:** 

	Milestone	Did not achieve level 1	Level 1	Level 2	Level 3
1	**Emergency Stabilization (PC1)**	□ Did not achieve Level 1	□ Recognizes abnormal vital signs	□ Recognizes an unstable patient, requiring intervention Performs primary assessment Discerns data to formulate a diagnostic impression/plan	□ Manages and prioritizes critical actions in a critically ill patient Reassesses after implementing a stabilizing intervention
2	**Performance of focused history and physical (PC2)**	□ Did not achieve Level 1	□ Performs a reliable, comprehensive history and physical exam	□ Performs and communicates a focused history and physical exam based on chief complaint and urgent issues	□ Prioritizes essential components of history and physical exam given dynamic circumstances
3	**Diagnostic studies (PC3)**	□ Did not achieve Level 1	□ Determines the necessity of diagnostic studies	□ Orders appropriate diagnostic studies. Performs appropriate bedside diagnostic studies/procedures	□ Prioritizes essential testing Interprets results of diagnostic studies Reviews risks, benefits, contraindications, and alternatives to a diagnostic study or procedure
4	**Diagnosis (PC4)**	□ Did not achieve Level 1	□ Considers a list of potential diagnoses	□ Considers an appropriate list of potential diagnosis May or may not make correct diagnosis	□ Makes the appropriate diagnosis Considers other potential diagnoses, avoiding premature closure
5	**Pharmacotherapy (PC5)**	□ Did not achieve Level 1	□ Asks patient for drug allergies	□ Selects an medication for therapeutic intervention, consider potential adverse effects	□ Selects the most appropriate medication and understands mechanism of action, effect, and potential side effects Considers and recognizes drug-drug interactions
6	**Observation and reassessment (PC6)**	□ Did not achieve Level 1	□ Reevaluates patient at least one time during case	□ Reevaluates patient after most therapeutic interventions	□ Consistently evaluates the effectiveness of therapies at appropriate intervals
7	**Disposition (PC7)**	□ Did not achieve Level 1	□ Appropriately selects whether to admit or discharge the patient	□ Appropriately selects whether to admit or discharge Involves the expertise of some of the appropriate specialists	□ Educates the patient appropriately about their disposition Assigns patient to an appropriate level of care (ICU/Tele/Floor) Involves expertise of all appropriate specialists
9	**General Approach to Procedures (PC9)**	□ Did not achieve Level 1	□ Identifies pertinent anatomy and physiology for a procedure Uses appropriate Universal Precautions	□ Obtains informed consent Knows indications, contraindications, anatomic landmarks, equipment, anesthetic and procedural technique, and potential complications for common ED procedures	□ Determines a back-up strategy if initial attempts are unsuccessful Correctly interprets results of diagnostic procedure
20	**Professional Values (PROF1)**	□ Did not achieve Level 1	□ Demonstrates caring, honest behavior	□ Exhibits compassion, respect, sensitivity and responsiveness	□ Develops alternative care plans when patients’ personal beliefs and decisions preclude standard care
22	**Patient centered communication (ICS1)**	□ Did not achieve level 1	□ Establishes rapport and demonstrates empathy to patient (and family) Listens effectively	□ Elicits patient’s reason for seeking health care	□ Manages patient expectations in a manner that minimizes potential for stress, conflict, and misunderstanding. Effectively communicates with vulnerable populations, (at risk patients and families)
23	**Team management (ICS2)**	□ Did not achieve level 1	□ Recognizes other members of the patient care team during case (nurse, techs)	□ Communicates pertinent information to other healthcare colleagues	□ Communicates a clear, succinct, and appropriate handoff with specialists and other colleagues Communicates effectively with ancillary staff

## INSTRUCTOR MATERIALS

**Case Title:** Managing Spontaneous Abortion Hemorrhage

**Case Description & Diagnosis (short synopsis):** A 33-year-old female presents with fatigue and active vaginal bleeding post-abortion, diagnosed with post-abortion hemorrhage (PAH) secondary to uterine atony. The ideal flow would be that the learners enter the room and find the patient in no acute distress, appearing fatigued. They immediately place the patient on the monitor, obtain the history of spontaneous abortion, and perform a physical exam, including GU exam, which will show active vaginal bleeding and a boggy uterus. Learners should recognize unstable vital signs, obtain IV access, send lab work, and obtain imaging which should include transvaginal or transabdominal ultrasound. The patient will then develop vaginal hemorrhage consistent with postpartum/post-abortion hemorrhage. Learners will be required to utilize physical maneuvers, initiate uterotonic medications, consider activating massive transfusion protocol (MTP), and consult OBGYN. Following administration of uterotonic medications, the patient’s vitals will improve but remain hypotensive with heavy vaginal bleeding. Learners will be prompted to demonstrate placement of a Bakri balloon. Once the Bakri balloon is placed, the patient will stabilize. OBGYN will arrive following this for planned surgical intervention and admission.

**Equipment or Props Needed:**

- Mannequins: Noelle and Victoria from Gaumard, draped with a bed sheet, placed in supine positioning with the head of the bed elevated to 45 degrees. Note that other complete or partial models could also be used if there is vaginal access into a simulated cervical or uterine model.
- Moulage: red-soaked Chux beneath mannequins (we used sugar-free red Jell-O).
- Bedside monitor with cardiac leads, blood pressure cuff, pulse oximeter, and end-tidal CO2.
- (2) 18 gauge IV with IV start kits
- Nasal/Oral airway and laryngeal mask airway
- 7.5 mm endotracheal tube with stylet
- Gum-elastic bougie
- Mac 4 laryngoscope blade with handle
- Nasal cannula or non-rebreather mask
- Bag-valve mask
- Suction tubing with canister
- Yankauer suction
- Bakri balloon kit with rapid instillation system, 500 mL normal saline bag for instillation (we emptied ours and filled with air because our Bakri balloons leak), and drainage bag.
- Foley catheter 16 French
- Medication vials labeled: methylergonovine, carboprost, tranexamic acid, oxytocin, and misoprostol

**Embedded participants needed:**

For the authors’ simulation, the facilitator voiced all parts of an intercom, but actors could be used to play parts of nurse, EMS, and OBGYN consultant.

**Stimulus Inventory:**

**Background and brief information:** The patient, a 33-year-old female, is brought to a tertiary care emergency department by EMS from a reproductive health clinic with complaints of fatigue and active vaginal bleeding.

**Initial presentation:**

- **Chief Complaint**: Fatigue and vaginal bleeding post-spontaneous abortion.
- **Vital Signs**:
  ○ Heart rate (HR): 123 bpm
  ○ Blood pressure (BP): 106/96 mmHg
  ○ Respiratory rate (RR): 18 breaths per minute
  ○ Temperature (T): 98.4 °F
  ○ Oxygen saturation (O2Sat): 98% on room air

**How the scenario unfolds:**

The case begins with a 33-year-old G2P1 female who presents via EMS from a reproductive health clinic for fatigue and vaginal bleeding at approximately 17 weeks’ gestation. She appears pale, fatigued, and mildly tachycardic on arrival.

If learners obtain a focused history, the patient reports heavy vaginal bleeding for several hours, soaking approximately one pad per hour, associated with cramping abdominal pain and passage of tissue. She denies fever or infectious symptoms. If asked, she confirms this is her second pregnancy and that her prior pregnancy was uncomplicated.

The patient will continue to bleed throughout the case unless appropriate hemorrhage control measures are initiated.

*Key events and critical actions include the following:*

### Event 1 – Initial presentation

Learners are expected to perform a primary survey, establish intravenous access, place the patient on cardiac monitoring, obtain a focused pregnancy history, and perform a GU examination. If a pelvic exam is performed, active bright red vaginal bleeding is noted. Ultrasound (if ordered) demonstrates heterogeneous material in the cavity of the uterus, likely to represent blood clots and no evidence of placental tissue, gestational sac, or yolk sac.

If pregnancy and hemorrhage are recognized early, the patient remains tachycardic but maintains perfusion. If learners fail to obtain pregnancy history, perform a GU exam, or order ultrasound, bleeding continues and the patient progressively decompensates, with worsening tachycardia and hypotension. She may verbalize increasing dizziness and weakness as a cue.

### Event 2 – Hemorrhagic Decompensation

If hemorrhage control measures are not initiated, the patient develops worsening hypotension and tachycardia.

Learners should administer uterotonic medications and activate massive transfusion protocol while consulting OBGYN. Learners may also choose to do physical maneuvers like uterine massage, bimanual compression, and manual clot evacuation, though these are not required for case progression. If uterotonics are administered, bleeding partially improves and vital signs stabilize but remain abnormal. This represents temporary control and requires escalation. If blood products are administered without uterotonics, hemodynamics do not meaningfully improve and bleeding continues. If neither uterotonics nor transfusion are initiated in a timely manner, the patient deteriorates into pulseless electrical activity arrest. Return of spontaneous circulation is achieved only if uterotonics are incorporated into resuscitation. Uterotonics that learners may consider using include oxytocin, misoprostol, methylergonovine, and/or carboprost.

### Event 3 – Escalation / Mechanical Intervention

Bleeding will persist despite above interventions and learners should escalate to mechanical intervention with placement of a Bakri balloon. If OBGYN is consulted, they are initially unavailable in person but can provide procedural guidance if requested. If the Bakri balloon is placed correctly, bleeding significantly decreases and hemodynamics improve. If mechanical control is not initiated, the patient remains unstable with risk of recurrent decompensation.

Following successful hemorrhage control, the patient stabilizes and OBGYN arrives to assume care. The case ends once hemorrhage is controlled and appropriate disposition is arranged.

**Critical actions:**

1. Place two large-bore IVs.
2. Perform a GU exam and obtain a transvaginal ultrasound.
3. Administer at least one uterotonic medication.
4. Consult OBGYN for guidance on further management.

**Case Title:** Managing Spontaneous Abortion Hemorrhage

**Chief Complaint:** Fatigue

**General Appearance:**

**Primary Survey:**

- **Airway:** Patent
- **Breathing:** Bilateral breath sounds
- **Circulation:** Tachycardic, pale

**History:**

- **History of present illness:** EMS, *if asked* for history, will state: “We received the call from a reproductive health clinic for a 33-year-old female. Patient was complaining of vaginal bleeding and feeling tired. Vitals were within normal limits.” Patient HPI: 32 y/o G2P1, 17 weeks pregnant, *if asked*, will state she spontaneously had vaginal bleeding and went to the reproductive health clinic where she was told she was having a miscarriage.
- **Past medical history:** Pre-eclampsia with prior pregnancy, otherwise uncomplicated, full-term.
- **Past surgical history:** none
- **Patient’s medications:** none
- **Allergies:** none
- **Social history:** none
- **Family history:** none

**Secondary Survey/Physical Examination:**

- **General appearance:** Fatigued appearing, otherwise WNL
- **HEENT:**
  ○ **Head:** WNL
  ○ **Eyes:** + Conjunctival pallor (must ask)
  ○ **Ears:** WNL
  ○ **Nose:** WNL
  ○ **Throat:** WNL
- **Neck:** WNL
- **Heart:** Tachycardic, regular
- **Lungs:** WNL
- **Abdominal/GI:** Gravid (must ask), mild suprapubic tenderness to palpation
- **Genitourinary:** Mild active vaginal bleeding, boggy uterus, cervical os is open, no lacerations (must ask each)
- **Rectal:** WNL
- **Extremities:** WNL
- **Back:** WNL
- **Neuro:** WNL
- **Skin:** Cool to touch
- **Lymph:** WNL
- **Psych:** Upset, tearful

#### Transvaginal ultrasound

IMPRESSION: Heterogeneous material in the cavity of the uterus, likely to represent blood clots. The uterus is enlarged without the presence of placental tissue, gestational sac, or yolk sac.

#### ECG

Lucinda Hampton. Physiopedia. Tachycardia.

https://www.physio-pedia.com/Tachycardia#/media/File:Achycardia_132_bpm.jpeg Published March 30, 2022. Accessed January 1, 2024. License CC BY-SA 3.0.

## OPERATOR MATERIALS

**Diagnosis:** Post-abortion hemorrhage secondary to uterine atony.

**Disposition:** Following stabilization with Bakri balloon placement, the patient should be admitted to the obstetrics/gynecology service for planned surgical intervention and ongoing monitoring.

## DEBRIEFING AND EVALUATION PEARLS

### Managing Spontaneous Abortion Hemorrhage

**Definition**^7^,^9^,^11^

- ≥500mL blood loss immediately after vaginal delivery
- ≥1000mL blood loss in 24 hrs or up to 12 weeks postpartum
- Bleeding with clinical signs/symptoms of hypovolemia within 24 hours of delivery

**Key Point:** Any abnormal vital sign should raise your suspicion for PPH.^5^,^7^,^9^

**Primary PPH:** Within first 24 hours consider: uterine atony, uterine rupture, laceration of the lower genital tract, retained placental tissue, uterine inversion, hereditary coagulopathy^7^,^9^,^11^

**Secondary PPH:** After first 24 hours consider: retained products of conception, uterine polyps, or coagulopathy^7^,^9^,^11^

**Basic Supportive Care**

- Large bore IV X 2, supplemental O2 if hypoxic, cardiac monitor^5^,^7^
- Volume expansion to replace hemorrhage^7^–^9^,^11^
  ○ Replace with blood products as soon as available
  ○ Give O negative until type specific products available
  ○ Can use crystalloid early if evidence of hypoperfusion but not ideal resuscitative fluid
- Call obstetrics or surgical consultants **early** because patients frequently require surgical intervention

**Common Causes (The “4Ts”) + Treatments**

- **T**one: Uterine Atony (the most common cause of PPH)^7^, ^9^,^11^
  ○ Bimanual Massage (external or internal/external).
  ○ Uterotonic Medications (give concomitantly with massage)
    ■ **Oxytocin** (1st line)^7^, ^9^, ^11^
      - Dose: 20 units/1 L NS IV at 250 mL/hr or 80 units/500 mL NS, IV run wide open or 10 units/hr (max: cumulative dose 40 units)
      - Intramuscular Dose: 10 units IM
    ■ **Misoprostol**^5^,^9^
      - Sublingual: 600 mcg sublingual (600–100 mcg)
      - Rectal: 1000 mcg (600–100 mcg)
    ■ **Methylergonovine**^7^,^9^,^11^
      - Dose: 0.2 mg IM, IV, or PO q 2–4 hours
      - Contraindicated in patients with preeclampsia or hypertension
    ■ **Carboprost**^7^,^9^,^11^
      - Dose: 250 mcg IM q 15–90 min (maximum dose 2 mg)
      - Contraindicated in patients with asthma
  ○ Bakri Balloon Tamponade (or Rusch Balloon)^7^,^10^,^11^
    ■ Insert through cervical os to the apex of uterine fundus (ultrasound may be useful in placement).
    ■ Fill device with 500 mL warm NS[Fig f3-jetem-11-3-s63]
- **T**rauma:
  ○ Uterine Inversion
    ■ Requires immediate manual reduction of uterus^7^,^9^,^11^
    ■ Discontinue uterotonic medications, start uterine relaxants^5^,^7^
      - Magnesium sulfate
        ○ Dose: 4–6 grams IV over 15 minutes
      - Terbutaline
        ○ Dose: 0.25 mg Sub Q or IV
      - Nitroglycerin
        ○ Dose: 50–250 mcg IV bolus over 1–2 min, then up to 3–4 additional doses q 3–5 min
        ○ Closely monitor blood pressure
  ○ Uterine Rupture: Requires surgery for correction^7^,^9^,^11^
- **T**issue: Retained Placental Tissue
  ○ Evaluate for any retained products (either manually or via US)^7^,^9^,^11^
  ○ Check to make sure the placenta is intact (no “missing pieces”)^7^,^9^
    ■ Remove either manually or via curettage
  ○ Consider Bakri or Rusch balloon tamponade^7^,^10^,^11^
- **T**hrombin, Disseminated Intravascular Coagulation (DIC)^7^–^9^,^11^
  ○ Look for oozing or ongoing bleeding from IV puncture sites
  ○ Reverse any coagulopathies
  ○ Replace blood products and coagulation factors
  ○ Operative intervention is likely necessary for hemorrhage control
- Consider administration of **tranexamic acid (TXA)** in all patients.^7^
  ○ Dose: 1000 mg IV over 10 minutes

Of note, some institutions are moving towards vacuum-induced hemorrhage control devices (e.g. JADA). Some data suggest less quantified blood loss, shorter device time, and potentially less blood product used with this device.^3^ Refer to your department protocols for device selection.

**Pearls**

- Post-abortion patients (spontaneous or medical/surgical) can experience significant bleeding and should be treated the same as patients with PPH.
- Strongly consider PPH in any patient with “more bleeding than expected” after vaginal delivery or abortion.
- Many patients with PPH will not have frank hemodynamic instability at initial presentation.
- All patients with PPH should have immediate consultation with obstetrics or general surgery (depending on your local resources) because many patients require operative intervention.
- Uterotonic medications should all be given together, in the absence of contraindications (i.e. do not wait for effect or no effect of medication before administering the next one).

**Other debriefing points:**

Anticipated management mistakes:

1. *Uncertainty about IF to place a Bakri balloon:* we modified our session to include prompting for balloon placement to ensure continued case flow.
2. *Uncertainty about how to place the Bakri Balloon:* We modified our session to include an introduction three months prior, which included a discussion of indications, contraindications, and placement of the Bakri balloon. Additionally, in the simulation case, we included the ability to have an OBGYN or facilitator walk the learner through its placement over the phone or intercom system.
3. *Uncertainty about which uterotonic medications to use:* We modified our session to reward learners for using ANY uterotonic medication. However, given their continued hemodynamic instability, this patient would likely benefit from administering all medications available.
4. *Failure to resuscitate with sufficient blood products:* we had one group unexpectedly favor the management of this patient’s hemorrhagic shock with pressor support. This led our patient to develop unstable supraventricular tachycardia **(**SVT) requiring synchronized cardioversion and ultimately prompting by nursing/facilitator to consider blood product administration.

## SIMULATION ASSESSMENT

*Managing Spontaneous Abortion Hemorrhage*

Learner: _________________________________________

***Assessment Timeline***

This timeline is to help observers assess their learners. It allows observer to make notes on when learners performed various tasks, which can help guide debriefing discussion.

**Critical Actions:**

□ Place two large-bore IVs.
□ Perform a GU exam and obtain a transvaginal ultrasound.
□ Administer at least one uterotonic medication.
□ Consult OBGYN for guidance on further management.

**Summative and formative comments:**

**Milestones assessment:**

## INSTRUCTOR MATERIALS

### Appendix A Pre- and Post-Intervention Learner Evaluation

Your participation in this survey is completely voluntary. Answers to the survey will remain anonymous to all parties involved. You may refuse to take part in the survey or exit the survey at any time without penalty. You are free to decline to answer any particular question you do not wish to answer for any reason.

In this first step, you will create a unique anonymous identifier for yourself. If you cannot answer any of the questions below, then leave it blank.

- What are the first two letters of the name of the high school you graduated from? _____
- What are the first two letters of the name of your college mascot? _____
- What are the first two letters of your mother’s maiden name? _____
- How many brothers do you have? _____
- How many sisters do you have? _____

**For the following statements, please indicate whether you strongly disagree, disagree, are neutral, agree, or strongly agree.**

1. I can **recognize** signs of postpartum/abortion hemorrhage.
  **Table:**
  Strongly Disagree	Disagree	Neutral	Agree	Strongly Agree
  1	2	3	4	5
2. I feel comfortable **managing** a case of postpartum/abortion hemorrhage.
  **Table:**
  Strongly Disagree	Disagree	Neutral	Agree	Strongly Agree
  1	2	3	4	5
3. I know what medications are available to me to treat postpartum/abortion hemorrhage.
  **Table:**
  Strongly Disagree	Disagree	Neutral	Agree	Strongly Agree
  1	2	3	4	5
4. I feel comfortable placing a Bakri balloon.
  **Table:**
  Strongly Disagree	Disagree	Neutral	Agree	Strongly Agree
  1	2	3	4	5

**Knowledge Test**

- Which postpartum/abortion hemorrhage medication is contraindicated in hypertension?
  - Carboprost
  - Methergine
  - Misoprostol
  - Oxytocin
- Which postpartum/abortion hemorrhage medication is contraindicated in asthma?
  - Carboprost
  - Methergine
  - Misoprostol
  - Oxytocin
- What is the maximum amount of fluid that should be instilled into the Bakri balloon?
  - 250 cc
  - 500 cc
  - 750 cc
  - 1000 cc
- Which of the following statements is **true** regarding the placement of a Bakri balloon?
  - The balloon can remain inflated safely for up to one week.
  - The device can be safely inflated if spanning across the internal and external cervical os.
  - This device is safe and effective in controlling hemorrhage in the setting of endometritis.
  - Placement of this device is contraindicated in the setting of placental retention.
- What is the most common cause of postpartum hemorrhage?
  - Placental retention
  - Laceration
  - Coagulopathy
  - Uterine atony

**Knowledge Test Answers**

- Which postpartum/abortion hemorrhage medication is contraindicated in hypertension?
  - Carboprost
  - **Methergine**
  - Misoprostol
  - Oxytocin
- Which postpartum/abortion hemorrhage medication is contraindicated in asthma?
  - **Carboprost**
  - Methergine
  - Misoprostol
  - Oxytocin
- What is the maximum amount of fluid that should be instilled into the Bakri balloon?
  - 250 cc
  - **500 cc**
  - 750 cc
  - 1000 cc
- Which of the following statements is **true** regarding the placement of a Bakri balloon?
  - The balloon can remain inflated safely for up to one week.
  - The device can be safely inflated if spanning across the internal and external cervical os.
  - This device is safe and effective in controlling hemorrhage in the setting of endometritis.
  - **Placement of this device is contraindicated in the setting of placental retention.**
- What is the most common cause of postpartum hemorrhage?
  - Placental retention
  - Laceration
  - Coagulopathy
  - **Uterine atony**
